# Two Coxsackievirus B3 outbreaks associated with hand, foot, and mouth disease in China and the evolutionary history worldwide

**DOI:** 10.1186/s12879-019-4107-z

**Published:** 2019-05-24

**Authors:** Zhenzhi Han, Yong Zhang, Keqiang Huang, Jianxing Wang, Huifang Tian, Yang Song, Qian Yang, Dongmei Yan, Shuangli Zhu, Mingxiao Yao, Xianjun Wang, Wenbo Xu

**Affiliations:** 1 WHO WPRO Regional Polio Reference Laboratory and National Health Commission Key Laboratory of biosafety, National Institute for Viral Disease Control and Prevention, Chinese Center for Disease Control and Prevention, No. 155, Changbai Road, Changping District, Beijing, 102206 People’s Republic of China; 2 Shandong Center for Disease Control and Prevention, Jinan City, Shandong Province People’s Republic of China; 3 Shijiazhuang Center for Disease Control and Prevention, Shijiazhuang City, Hebei Province People’s Republic of China; 4 Anhui University of Science and Technology, Hefei City, Anhui Province People’s Republic of China

**Keywords:** Enterovirus, CV-B3, Molecular epidemiology, HFMD

## Abstract

**Background:**

Coxsackievirus B3 (CV-B3) is usually associated with aseptic meningitis and myocarditis; however, the association between CV-B3 and hand, foot, and mouth disease (HFMD) has not been clearly demonstrated, and the phylogenetic dynamics and transmission history of CV-B3 have not been well summarized.

**Method:**

Two HFMD outbreaks caused by CV-B3 were described in Hebei Province in 2012 and in Shandong Province in 2016 in China. To analyze the epidemiological features of two CV-B3 outbreaks, a retrospective analysis was conducted. All clinical specimens from CV-B3 outbreaks were collected and disposed according to the standard procedures supported by the WHO Global Poliovirus Specialized Laboratory. EV genotyping and phylogenetic analysis were performed to illustrate the genetic characteristics of CV-B3 in China and worldwide.

**Results:**

Two transmissible lineages (lineage 2 and 3) were observed in Northern China, which acted as an important “reservoir” for the transmission of CV-B3. Sporadic exporting and importing of cases were observed in almost all regions. In addition, the global sequences of CV-B3 showed a tendency of geographic-specific clustering, indicating that geographic-driven adaptation plays a major role in the diversification and evolution of CV-B3.

**Conclusions:**

Overall, our study indicated that CV-B3 is a causative agent of HFMD outbreak and revealed the phylogenetic dynamics of CV-B3 worldwide, as well as provided an insight on CV-B3 outbreaks for effective intervention and countermeasures.

**Electronic supplementary material:**

The online version of this article (10.1186/s12879-019-4107-z) contains supplementary material, which is available to authorized users.

## Background

Coxsackievirus B3 (CV-B3), which belongs to the genus *Enterovirus* and family Picornaviridae, is an important pathogen that causes several infectious diseases, ranging from a mild febrile syndrome or respiratory illness to aseptic meningitis, myocarditis, and encephalitis [1, 2]. Since 1987, when the full-length genome of CV-B3 was first reported, several outbreaks caused by CV-B3 were reported in different parts of the world [3]. Repeated outbreaks of aseptic meningitis and myocarditis caused by CV-B3 have been frequently reported [4–6]. Furthermore, the epidemiological data and incidence of CV-B3 in infants and children have also been investigated in a prospective cohort study in Jiangsu Province, China [7]. In Yantai city of China, a seroprevalence study of CV-B3 indicated that children aged < 5 years were the most susceptible population and that CV-B3 is widely distributed in the population of children, with a seroprevalence of 52.3% [8]. CV-B3 was also isolated from cases of acute flaccid paralysis in India and underwent frequent recombination with other enteroviruses [9]. In Spain, neurological and respiratory diseases caused by CV-B3 were detected in 10% of positive myocarditis cases from 2004 to 2014, including two fatal myocarditis cases [10]. In the United States, 5.4% of fatal cases were associated with CV-B3 infections during 1970–2005 [11], whereas enterovirus surveillance studies in other countries, such as Germany and France, reported the prevalence of CV-B3 between 1 and 6.5%, depending on the year and country [10, 12, 13].

However, other molecular epidemiological reports of CV-B3 were only on the sporadic detection of CV-B3 in China and other Asian countries and did not systematically summarize the evolutionary and transmission history in the world. Other reports on phylogenetic analysis using the partial *VP1* coding region did not clearly account for the evolutionary dynamics and epidemiological characteristics of CV-B3 [10, 14]. Several studies have shown that CV-B3 is associated more with aseptic meningitis, myocarditis, and mild flu-like illness, while only a few studies definitely demonstrated the association between CV-B3 and hand, foot, and mouth disease (HFMD); however, these did not help us clearly understand the epidemiological characteristics to prevent this disease. This study aimed at overcoming the limitations of previous studies by providing a clear interpretation.

In the summer of 2012 and 2016, two outbreaks of CV-B3 infections associated with HFMD were detected in Hebei and Shandong Province, China, respectively. Although the number of cases were less than the patients infected by enterovirus A71 (EV-A71), coxsackievirus A16 (CV-A16) and coxsackievirus A6 (CV-A6) [15–23], genetically linked CV-B3 were detected in HFMD patients involved in outbreaks, suggesting a possible association between CV-B3 infection and HFMD. In this study, the outbreak investigation provides insight into CV-B3 epidemiology and evolutionary history of CV-B3.

## Methods

### Ethics approval

In this study, the only human materials used were clinical samples, including stool samples, throat swabs, and anal swabs collected from HFMD patients at the investigation of the National Health Commission of the People’s Republic of China for public health purposes. Written informed consent for the use of their clinical samples was obtained from the parents of the children whose samples were analysed by signing the informed consent when collecting the samples. In brief, when investigators collected the clinical samples in hospital, the use of their clinical samples was explained for the guardians of children and written consent was signed by guardians of children for permitting analysis of their clinical samples. At this procedure, the staff of National Health Commission of the People’s Republic of China confirmed the truth that the guardians of children fully understood the use of their clinical samples. Finally, the written consent was delivered to the study co-ordinatorsby the investigators under the surveillance of local institute of health Commission. The study co-ordinators performed the analysis of clinical samples for the surveillance of enterovirus and public health purposes. This study was approved by the Second Ethics Review Committee of the National Institute for Viral Diseases Control and Prevention, Center for Disease Control and Prevention, China.

### Patients and sample collection

Based on the National HFMD pathogen surveillance system which was built in 2008 in the mainland of China, the HFMD cases were reported and the representative samples were sent to the national HFMD laboratory for enterovirus-confirmed. We defined a probable HFMD patient as a patient who had rashes on the hands, feet, mouth, or buttocks and ulcers or vesicles in the mouth with or without fever. We defined a laboratory-confirmed patient as a probable patient with laboratory evidence of infection with EV-A71, CV-A16, or other enteroviruses. The diagnostic tests used for CV-B3 detection were reverse transcription PCR (RT-PCR) and real-time reverse transcription PCR (qRT-PCR), as described previously [7, 24, 25]. Patients were classified as having severe HFMD if they had any complications (e.g. aseptic meningitis, brainstem encephalitis, encephalitis, encephalomyelitis, acute flaccid paralysis or autonomic nervous system dysregulation, pulmonary edema, pulmonary hemorrhage, or cardiorespiratory failure). Otherwise, patients were classified as having mild HFMD. It is consistent with the regulations of national HFMD. 50% of clinical samples were randomly selected for nucleic acid testing every month, with the maximum was 30. A total of 36 clinical samples were randomly harvested from these two outbreaks, including 18 clinical samples collected from the Hebei Province in 2012 and 18 clinical samples collected from the Shandong Province in 2016. Eighteen CV-B3 strains were isolated from stool specimens collected from 18 HFMD patients during an outbreak in Shijiazhuang City of Hebei Province in 2012 and another 18 CV-B3 strains were isolated from stool specimens collected from 18 HFMD patients during an outbreak in Shandong Province in 2016. The stool samples were processed according to the standard procedures and were then inoculated onto RD cells provided by the WHO Global Poliovirus Specialized Laboratory for viral isolation. Infected cell cultures were harvested after complete cytopathic effect was observed.

### Sequencing and molecular typing

Viral RNA was extracted from the cell cultures using the QIAamp Viral RNA Mini Kit (Qiagen, Germany). Reverse transcription PCR was performed to amplify the complete *VP1* coding region using the PrimeScript One Step RT-PCR Kit Ver.2 (TaKaRa, Dalian,China) with primers 490–493 [25]. The amplicons were sequenced using ABI 3130 Genetic Analyser (Applied Biosystems, Foster City, CA, USA) to harvest the complete *VP1* region. The acquired *VP1* sequences were analyzed with the BLAST server by comparing the identity of sequences available in the GenBank and were determined using the EV Genotyping Tool [26].

### Phylogenetic analysis

The evolutionary history of CV-B3 was studied by maximum likelihood analysis and Bayesian inference method. A total of 236 entire *VP1* nucleotide sequences (dated to December 2017) with known sampling dates in the world were selected for phylogenetic analysis, including sequences in this study and sequences incorporated from GenBank (Additional file 1: Table S1). To investigate the epidemiological pattern in the mainland of China, 134 entire *VP1* nucleotide sequences were used to analyze the phylogenetic characteristics. Nucleotide sequences were aligned with their corresponding homology by Muscle implanted in MEGA software (version 7.0.26) [27]. The maximum likelihood phylogenetic tree was constructed by the IQ-TREE software and inferred by ModelFinder to search the best nucleotide substitution model of GTR + F + Γ_3_, including the General Time Reversible model (GTR), rate heterogeneity of Gamma distribution with rate categories of 3 (Γ_3_) and Empirical base frequencies (F) [28, 29]. Phylogenetic trees were also inferred by using Bayesian method implemented in BEAST software (version 1. 7.5) [30], with the nucleotide substitution model of GTR + I + Γ supported by the jModelTest software (version 2) [31]. The topology of phylogenetic trees was also assessed using the MrBayes software (version 3. 2. 6) and RaxML software (version 8) to confirm the topology of phylogenetic trees [32, 33]. The Markov Chain Monte Carlo chain was run for 1.5 × 10^8^ generations to establish convergence of all parameters. Convergence and effective sample size (> 200) of the parameters were checked with Tracer software (version 1.6) [34]. The resulting trees were summarized using a maximum clade credibility (MCC) topology from TreeAnnotator software (version 1.8.4), with a burn-in of the first 10% of sampled trees. We used the FigTree software (version 1.4.2) to manipulate the phylogenetic trees for the best performance. Sampling times of the sequences were used to calibrate the molecular clock. We performed date randomization tests in R package (version 3.4.3) using the Tip Dating Beast package to determine the temporal signal in the data [35]. Based on 20 random replicates of the sampling dates produced by this package, the datasets are considered to have sufficient temporal signal for the datasets when the 95% credibility intervals of rate estimate of real datasets doesn’t fall within the 95% credibility intervals of rate estimate from the date randomized replicates. This approach can provide a more accurate test for the temporal structure of CV-B3 datasets so that we could accurately estimate the evolutionary timescale of CV-B3. A Bayes factor analysis was performed to select the best demographic model and compare different models for the best one.

After the CV-B3 sequences had sufficient temporal signals, the gene timing of origin was calculated, which added a timescale to the phylogenetic histories, and the their most recent common ancestors (tMRCA) were calculated [36], based on a relaxed uncorrelated exponential growth coalescent inference and a relaxed uncorrelated lognormal growth coalescent inference. To determine the extent to which the viral population was constructed by geography, phylogeny-trait association analysis was performed using BaTS software (version 2.0) to compute the values of the association index, parsimony score, and maximum monophyletic clade statistics [37]. *P* values of < 0.05 were considered significant from the three statistics. Natural selection pressure on the entire *VP1* region of CV-B3 was assessed by estimating the ratio of nonsynonymous substitution to synonymous substitution implemented in the software of PAML 4.7 [38] and on-line Datamonkey [39, 40]. Likelihood ratio tests of the former were performed to compare these nested models (M0 vs. M3, M1a vs. M2a, M7 vs. M8) for selecting the one that fitted the data best. The latter, which used the methods of MEME (Mixed Effects Model of Evolution) and FEL (Fixed Effects Likelihood), was considered to be under the positive selection with *p* < 0.05.

### Nucleotide sequence accession numbers

The nucleotide sequence of the entire *VP1* gene for all strains, which was determined in this study, has been deposited in the GenBank nucleotide sequence database under accession number MH293510-MH293534.

## Results

### Two HFMD outbreaks associated with CV-B3

**Fig. 1:**
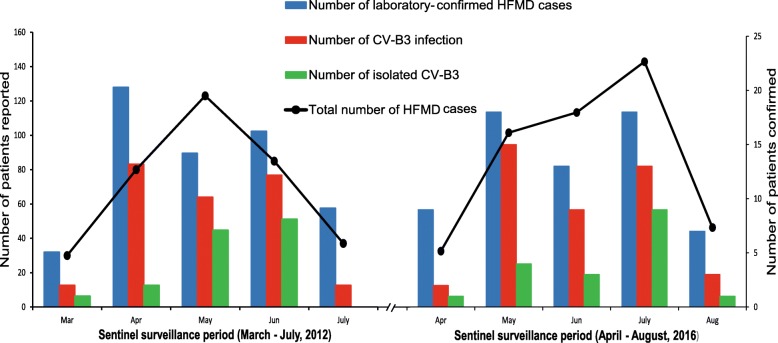
The number of HFMD cases reported and laboratory-confirmed cases of HFMD and CV-B3 infection. The left panel show the sentinel surveillance data of Shijiazhuang City of Hebei Province, from March to July, 2012. The right panel show the sentinel surveillance data of Shandong Province, from April to August, 2016. The line chart represent the total number of HFMD cases corresponding to the left vertical coordinates. The bar graph represent the laboratory-confirmed cases of HFMD and CV-B3 infection corresponding to the right vertical coordinates

From March to July of 2012, the CV-B3 caused a small-scale outbreak of HFMD in the Shijiazhuang City of Hebei Province, China, with the peak morbidity during the April and July (Fig. 1). A total of 355 HFMD cases were reported in the surveillance system and 64 patients with HFMD were confirmed by the laboratory diagnosis. 35 cases with the CV-B3 infection were further confirmed in the lab test described in the section of Methods. 50% of clinical samples were randomly selected for nucleic acid testing every month, with the maximum was 30. A total of 18 strains were successfully isolated and serotyped for next analysis. Based on the number of the total HFMD cases reported, about 9.9% of the pathogens spectrum is the serotype of CV-B3. And the patients’ number of CV-B3 infection occupy 54.7% proportion of all laboratory-confirmed HFMD cases. All children, whose clinical samples were successfully isolated, lived in the Shijiazhuang City of Hebei Province and presented the common disease of HFMD (e.g. rashes on the hands, feet, mouth, or buttocks and ulcers or vesicles in the mouth) when they were diagnosed.

Another small-scale outbreak of CV-B3 occurred in Shandong Province in 2016 (Fig. 1). A total of 443 HFMD patients were reported from April to August of 2016 and 65 patients were laboratory-confirmed HFMD cases. In the laboratory test, 42 clinical samples of HFMD patients were positive to the CV-B3 detection. The number of CV-B3 infection account for 64.6% of all laboratory-confirmed HFMD cases. The clinical samples were randomly selected about 10 samples each month at the process of outbreak for further serotyping and sequencing. And the laboratory successfully isolated 18 CV-B3 strains from the clinical samples of these children. To the children whose clinical samples were successfully isolated, they presented the mild symptoms of HFMD (e.g. rashes on the hands, feet, mouth, vesicles in the mouth with or without fever) when they were diagnosed in hospital. The similar symptom, high proportion of CV-B3 detection during the outbreak, close phylogenetic association and CV-B3 diffusion in a short timescale show the association between the CV-B3 and the occurrence of the outbreak.

A total of 36 strains, including 18 Hebei strains (collected in 2012) and 18 Shandong strains (collected in 2016), which accounted for two HFMD outbreaks, were isolated. Specimens collected from these 36 children consisted of 30 fecal samples, 5 throat swabs and 1 anal swab. Epidemiological investigation showed that most of the children were aged < 5 years (*n* = 34, 94.4%), ranging from 3 months to 7 years. In these children, comprising 22 boys and 14 girls, the most common disease presented were HFMD. The major timescale of sampling were observed in May (*n* = 11, 30.5%), June (n = 11, 30.5%) and July (*n* = 9, 25%).

### Molecular epidemiology of CV-B3 in China

**Fig. 2:**
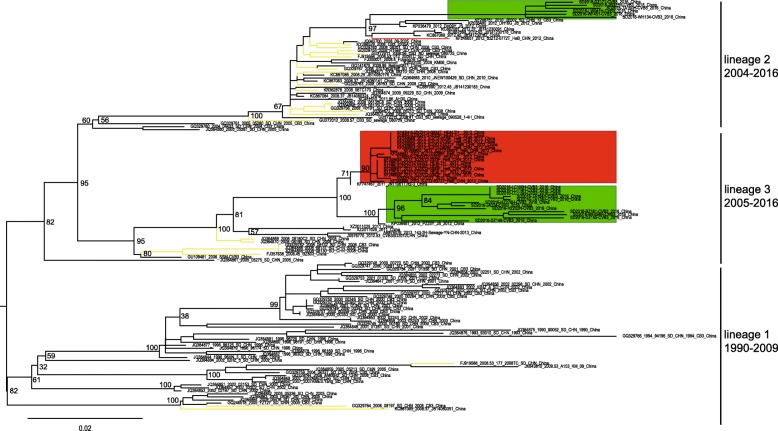
Midpoint-rooted maximum likelihood phylogenetic trees of the 135 coxsackievirus B3 (CV-B3) isolates from mainland China. The scale bars represent the substitutions per site per year. The three lineages are named according to appearing timescale of the isolated strains. The green module represents the CV-B3 strains isolated in Shandong Province in 2016. The strains isolated in Hebei Province in 2012 are colored in red. The branches colored in yellow represent the CV-B3 strains isolated from the outbreak of 2008

The midpoint-rooted maximum likelihood phylogenetic trees showed that three lineages (lineage 1–3) circulated in the mainland of China (Fig. 2). The lineage 1, circulating from 1990 to 2009, comprised mostly CV-B3 strains isolated from Shandong Province and tended to disappear in recent years. However, the CV-B3 strains in lineage 2 and lineage 3 evolved rapidly and caused outbreaks in recent years. The lineage 2, circulating in mainland of China from 2004 till present, consisted of many strains isolated from three outbreaks (aseptic meningitis outbreak in 2008, HFMD outbreaks in 2012 and 2016). The lineage 3, which circulated in mainland of China from 2005 till present, also included many strains isolated from the above three outbreaks. In 2008, an outbreak of aseptic meningitis mainly caused by the lineage 2 of CV-B3 occurred in Shandong Province [41] (Fig. 2, colored in yellow). This outbreak had also spread to other regions of China, as seen by the similar strains that were also isolated from Yunnan Province, Fujian Province and Shenzhen City though these three regions are distant. The strains of 08–2035 (GenBank accession number JQ042700) and JB14080176 (GenBank accession number KC867086), which were isolated from Fujian Province and Shenzhen City respectively, cluster with the strains of CV-B3 isolated from Shandong Province in 2008 directly. The strain of KM06 (GenBank accession number KJ020100), which was isolated from Yunnan Province in 2009, was encompassed by other strains of Shandong Province. It is indicated that the strain of KM06 possibly originated from the Shandong Province through the population movements. The small-scale outbreak of HFMD in 2012 in Hebei Province was mainly caused by lineage 3 of CV-B3 (Fig. 2, colored in red). Similar to the outbreak in 2012, the lineage 2 and lineage 3 caused a small-scale HFMD outbreak in 2016 in Shandong Province (Fig. 2, colored in green).

### Global groups distribution of CV-B3

**Fig. 3:**
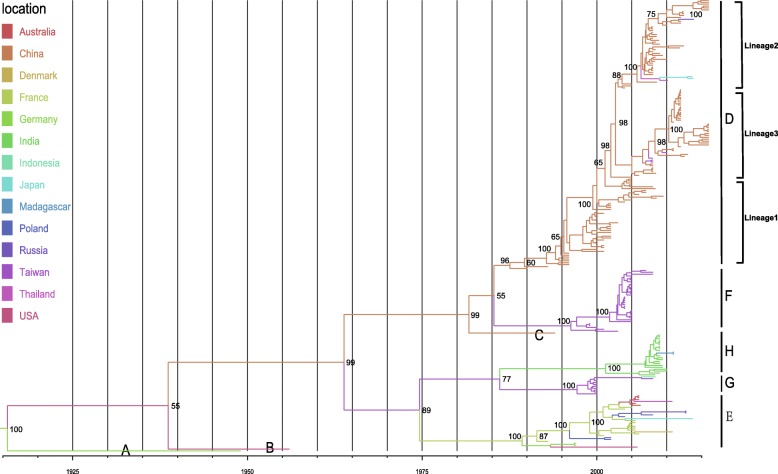
Maximum clade credibility (MCC) phylogenetic tree for the entire *VP1* sequences of coxsackievirus B3 (CV-B3) in the world. The branches were colored according to the location of isolates. The isolates were classified into 8 groups, shown in the figure, denoted A to H (names based on the dates in order of appearance of groups). The detailed information of isolates, including GenBank accession numbers and names of strains, are presented in Additional file 1: Table S1

The MCC tree of 236 entire *VP1* sequences of CV-B3, sampled from 1949 to 2016, classified all the CV-B3 sequences into 8 groups, A–H (Fig. 3). To avoid the bias of Bayesian phylogenetic inference, the maximum likelihood phylogenetic tree was also constructed to compare the topology identity of these two phylogenetic tree. And distinct difference was not observed by these two methods. The names of groups were based on the dates in order of appearance of groups and genetic distance described below. The mean genetic distance between the eight groups, which was calculated using the Kimura 2-parameter model, is ranging from 12.6 to 31.6% and is larger than the mean genetic distance within the eight groups (ranging from 3.3 to 9.4%), indicating the reliability of genotyping. The data included in the Bayesian analysis passed the date randomization tests showing no overlaps between the 95% credibility intervals (CIs) of rate estimate of real datasets and 95% CIs generated from 20 replicates of date randomization (Additional file 1: Figure S1). Phylogenetic analysis illustrated that 5 groups (D–H) still circulates throughout the world, whereas groups (A–C) have disappeared in the last century. Moreover, each CV-B3 group was not located in one region only, showing a global distribution pattern. Group D has been persistently evolving and being transmitted in the mainland of China, tending to develop three major subgroups. In the mid-1980s, the group D was formed and initially detected in 1990 in Shandong Province of China [41]. Sporadic exporting of cases that originated from the mainland of China, such as two strains from Japan, one each strain from Thailand and Russia were monitored. The strains in the group E, which included many sequences that originated from European countries, persistently circulated in the European regions. Within the group E, strains sampled from the different regions were observed, showing a certain level of geographical gene flow, whereas most gene groups may persist in a particular location, with occasional movement of viral lineages among locations. Groups F and G, which circulated in Taiwan of China, have circulated for about 20 years and caused an outbreak in 2012 (unfortunately, not all entire *VP1* sequences could be obtained from GenBank databases for this analysis) [42]. Group H comprised a number of sequences isolated from acute flaccid paralysis cases in India. Furthermore, the CV-B3 strains in India were more likely to recombine with other serotypes of enterovirus, such as enterovirus B74 from the Tibet Autonomous Region of China [9].

### Evolutionary characteristics of CV-B3 groups

To understand the evolutionary dynamics of CV-B3, we estimated the dates of origin of each group using a Bayesian relaxed molecular clock method. We calculated the evolutionary rate of the *VP1* sequence to be 6.19 × 10^− 3^ substitutions per site per year (95% HPD, 4.77–7.56) (Additional file 1: Table S2). The results indicated substantial heterogeneity in evolutionary rates among different lineages, with an estimated coefficient of variation of 0.9153 (95% HPD, 0.8358–0.9915), suggesting that relaxed molecular clock was more suited. Using the estimated molecular clock, the common ancestor of group D was dated to about 1984 (95% HPD, 1978–1988) when it was first isolated in 1990 in China. Group F and G have emerged in 1995 (95% HPD, 1991–1998) and 1996 (95% HPD, 1992–1999), respectively. The group E was dated to 1989 (95% HPD, 1984–1992), whereas the group H have emerged in 2000 (95% HPD, 1993–2004) before its first isolation in the world. The various groups were identified approximately 4 to 8 years after their dates of common ancestry.

Except for some insufficient samples, the isolates tended to cluster according to their geographical origin. Although the MCC phylogenetic tree showed a trend in geographical cluster (Fig. 3), the association index (*p* < 0.001) and parsimony score values (p < 0.001) clearly showed that the CV-B3*VP1* sequence was more phylogenetically clustered by regions. The maximum monophyletic clade of all regions showed high scores (*p* < 0.01). We observed the significant feature of geographical structure when the isolates were grouped by geographical origin (Table 1). The phylogeny-trait association test provided further evidence that the diversification of CV-B3*VP1* sequences were accounted for by geographical-driven adaptation. In addition, no positive selection positions were detected using the method of phylogenetic analysis by maximum likelihood. However, the purifying selection was detected at the majority of polymorphic sites by datamonkey analysis, illustrating that many mutations in *VP1* sequence of CV-B3 were harmful and consequently eliminated by natural selection.

**Table 1:** Analysis of the geographical structure of coxsackievirus B3 (CV-B3) strains

Statistic	Isolates	Observed mean(95%HPD)	Null mean(95%HPD)	Significance
AI		2.06 (1.70,2.43)	16.75 (15.32,17.98)	< 0.001^***^
PS		22.86 (22,24)	97.24 (94.01,100.68)	< 0.001^***^
MC (Mainland of China)	135	31.75 (31,33)	5.15 (3.72,7.21)	0.00999999^**^
MC (France)	11	5.03 (5,5)	1.14 (1,2)	0.00999999^**^
MC (USA)	2	N/A	N/A	N/A
MC (Australia)	5	2.33 (2,3)	1.02 (1,1.19)	0.02^*^
MC (Denmark)	1	N/A	N/A	N/A
MC (Taiwan of China)	46	33 (33,33)	2.19 (1.6,3)	0.00999999^**^
MC (India)	20	9.63 (9,13)	1.41 (1,2.01)	0.00999999^**^
MC (Germany)	3	2 (2,2)	1.01 (1,1)	0.00999999^**^
MC (Indonesia)	1	N/A	N/A	N/A
MC (Madagascar)	2	N/A	N/A	N/A
MC (Poland)	4	2 (2,2)	1.02 (1,1.11)	0.00999999^**^
MC (Thailand)	2	N/A	N/A	N/A
MC (Russia)	3	2 (2,2)	1.001 (1,1)	0.00999999^**^
MC (Japan)	3	2 (2,2)	1.004 (1,1)	0.00999999^**^

*AI* association index, *PS* parsimony score, *MC* maximum monophyletic clade, *HPD* highest probability density interval;

N/A, no data available due to insufficient sample size (*n* < 3);

Significance thresholds: *, 0. 01 < *p* < 0. 05; **, 0. 001 < *p* < 0. 01; ***, *p* < 0. 001

## Discussion

It is well known that HFMD is a common infectious disease in young children, especially those aged < 5 years [43, 44]. The most common etiological agents of HFMD are EV-A71 and CV-A16, which were reported and confirmed in various studies [15, 16, 21, 45–48]. Except for EV-A71 and CV-A16, CV-A6 and CV-A10 were gradually confirmed as pathogens that caused large-scale outbreaks of HFMD worldwide [17, 20, 23, 49]. However, CV-B3 was not confirmed as a major pathogen responsible for outbreaks of HFMD, although it was usually reported as a pathogen causing the outbreaks of aseptic meningitis and myocarditis [6, 10, 14, 41, 50, 51]. In this study, two HFMD outbreaks associated with CV-B3 were confirmed, and it has been proved that CV-B3 is a pathogen that causes HFMD. Genetically linked CV-B3 was identified in HFMD patients and healthy individuals, which is in line with the characteristics of enterovirus infection and transmission. The different levels of outbreaks associated with CV-B3 in China, which formed the peak in 2000–2002 (aseptic meningitis), 2004–2005 (aseptic meningitis), 2008 (aseptic meningitis), and 2012 (symptoms related to enterovirus infection), were also reported in addition to the two outbreaks described in this study [14, 41, 42]. Different areas, though far away, might experience CV-B3 outbreaks over a similar timescale, such as the Shandong Province and Hong Kong of China experienced the similar aseptic meningitis outbreaks in 2008.

The analysis of entire *VP1* sequence in mainland of China illustrated that lineage 2 and lineage 3 persistently circulated and evolved in North China (Hebei Province and Shandong Province). However, the number of entire *VP1* sequences of CV-B3 collected in GenBank was very few due to possibly incomplete surveillance of enteroviruses worldwide and unreported studies of CV-B3. Although the bias caused by the above-mentioned factors existed, we tried our best to collect all the entire *VP1* sequences of CV-B3 from GenBank and analyze the possible transmissible routes of CV-B3. The clusters of *VP1* sequences of CV-B3 indicated that Shandong Province acted as a ‘reservoir’ for transmission of CV-B3, such as the CV-B3 isolates of Shandong Province clustered with strains isolated from Yunnan Province of China (Fig. 2). We found that the CV-B3, which was isolated from the outbreak of HFMD in 2012 and 2016, evolved from the outbreak of the 2008 aseptic meningitis. Fortunately, the symptoms of HFMD patients caused by CV-B3 infection were all mild, and no severe cases or deaths were reported.

Global entire *VP1* sequences of CV-B3, available from GenBank and our databases, were analyzed through Bayesian phylogenetic inference. The MCC tree of CV-B3 based on entire *VP1* sequences showed that all the strains were clustered into 8 divergent groups (Fig. 3). Sporadic exporting and importing of cases were observed in almost all regions, indicating a tendency of globalization of infectious diseases. A ladder-like tree structure, sampling in different timescales and locations, showed that the virus evolved over time. Isolation of enteroviruses in cell culture, followed by serotyping based on a neutralization assay, is regarded as the recommended method for diagnosing enteroviral infections before the era of molecular serotyping arrives. In the GenBank, nearly half of the entire *VP1* sequences of CV-B3 were from China. One of the reasons may be the improvement of enterovirus surveillance in mainland of China so that more CV-B3 strains have been sequenced. Another possible reason is that CV-B3 is very prevalent in China. Therefore, this could likely cause some bias in analyzing the phylogenetic dynamics. However, we cannot obtain more data on CV-B3 from the public databases to analyze the epidemiological characteristics. Although the sample size is low, the collective outbreak and close phylogenetic association show the evidence between the isolation of CV-B3 and the occurrence of outbreak. More enterovirus surveillances must be perfected to trace the evolutionary dynamics of enterovirus, responding to the appeal from European scientists [52]. Current surveillance and basic research efforts must be strengthened for understanding viral pathogenesis and developing effective medical countermeasures [53].

Except for some strains with insufficient sample size, most global CV-B3 strains showed a tendency of geographical clustering. The results of phylogenetic trait association analysis provided evidence for geographical clustering of CV-B3 isolates from nine countries. It is speculated that geographically driven adaptation was responsible for the CV-B3 diversification. But we also realized that the geographical clustering of CV-B3 global isolates should be illustrated cautiously. Due to unavailable data of most hosts, the possible role of host-driven adaptation was not investigated. We need further studies to investigate possible host-driven adaptation of CV-B3 sequences.

## Conclusion

In summary, this study is the first to confirm that CV-B3 can cause HFMD outbreaks and also provides insight into the evolutionary history of CV-B3 worldwide. The pathogen spectrum of HFMD was amplified again so that we could more clearly understand HFMD. We found that genetic variations were correlated with geographical regions. These results have increased our knowledge about the evolution and outbreaks of CV-B3 and can help in developing sustainable management strategies to control this virus.

## Additional file

Additional file 1: **Figure S1.** The result of date-randomization tests (DRTs). The temporal signal of CV-B3 datasets was tested using the Tip Dating Beast package. Based on 20 random replicates of the sampling dates produced by this package and the real datasets, the CV-B3 datasets are assured to have sufficient temporal signals for next assessment of evolutionary timescale. **Table S1.** The information of 236 coxsackievirus B3 (CV-B3) strains used in this analysis, including 25 isolates first reported in this study. **Table S2.** Evolutionary characteristics of coxsackievirus B3 (CV-B3) groups based on the entire *VP1* gene. (DOCX 165 kb)

## Data Availability

The nucleotide sequence of the entire *VP1* region, which was determined in this study, has been deposited in the GenBank under accession number MH293510-MH293534.

## Abbreviations

CV-B3: Coxsackievirus B3
HFMD: Hand, foot, and mouth disease
tMRCA: The their most recent common ancestors
