# Supplementary material for: Two Coxsackievirus B3 outbreaks associated with hand, foot, and mouth disease in China and the evolutionary history worldwide
Source: BMC Infect Dis. 2019 May 24;19:466. doi: 10.1186/s12879-019-4107-z (PMC6534883; doi:10.1186/s12879-019-4107-z)
Supplement: Supplementary file 1 — Figure S1. The result of date-randomization tests (DRTs). The temporal signal of CV-B3 datasets was tested using the Tip Dating Beast package. Based on 20 random replicates of the sampling dates produced by this package and the real datasets, the CV-B3 datasets are assured to have sufficient temporal signals for next assessment of evolutionary timescale. Table S1. The information of 236 coxsackievirus B3 (CV-B3) strains used in this analysis, including 25 isolates first reported in this study. Table S2. Evolutionary characteristics of coxsackievirus B3 (CV-B3) groups based on the entire VP1 gene. (DOCX 165 kb) [file 12879_2019_4107_MOESM1_ESM.docx]

Fig S1. The result of date-randomization tests (DRTs). The temporal signal of CV-B3 datasets was tested using the Tip Dating Beast package. Based on 20 random replicates of the sampling dates produced by this package and the real datasets, the CV-B3 datasets are assured to have sufficient temporal signals for next assessment of evolutionary timescale.


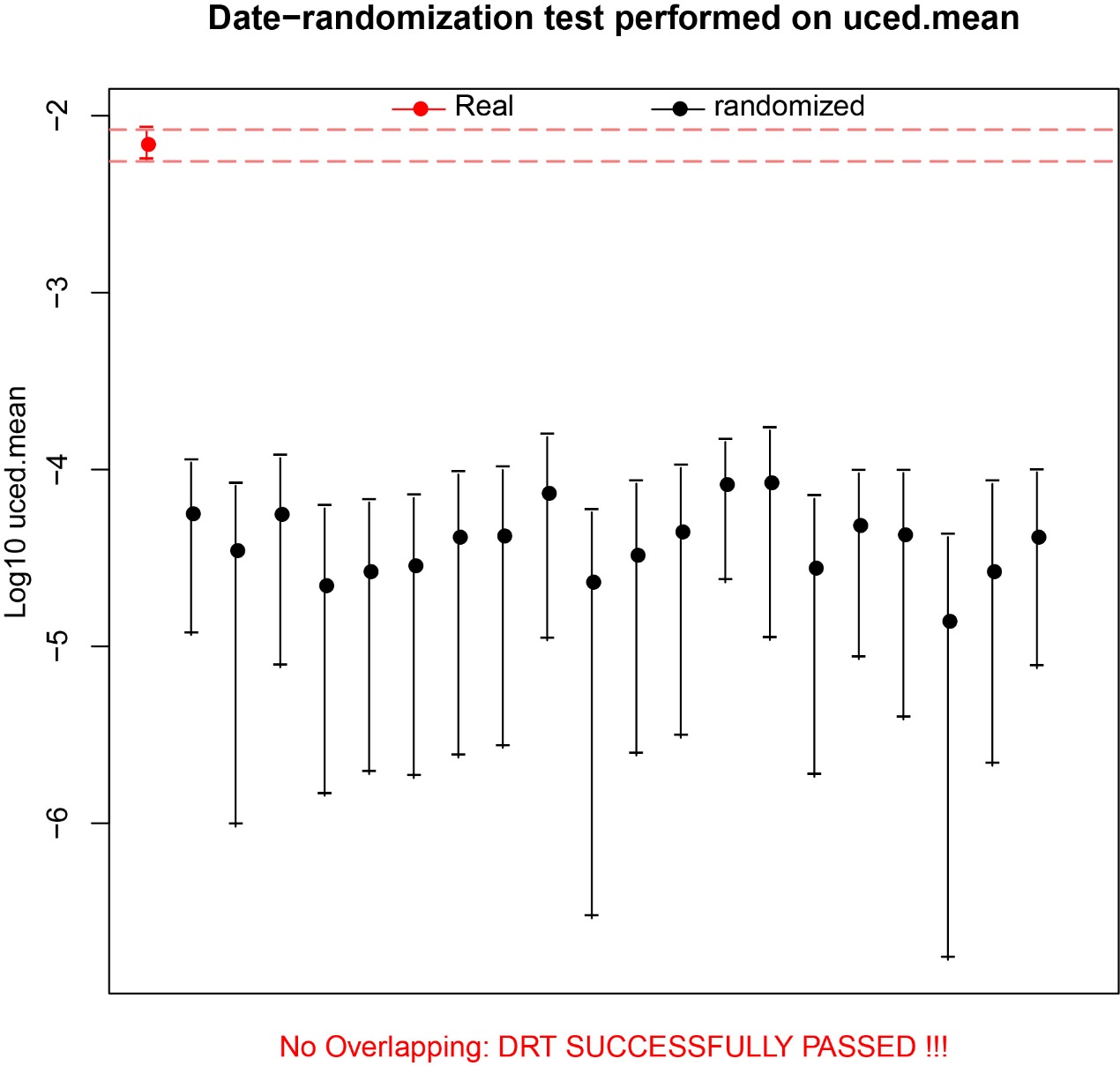


Table S1. The information of 236 coxsackievirus B3 (CV-B3) strains used in this analysis, including 25 isolates first reported in this study.

| Isolation year | Countries or regions | Strain name | GenBank accession No. | Origin |
| --- | --- | --- | --- | --- |
| 2013 | China | 143-2H-Sewage-YN-CHN-2013 | AB976076 | GenBank |
| 2005.5 | France | CF1851041-05 | AM236933 | GenBank |
| 2005.5 | France | CF1880311-05 | AM236936 | GenBank |
| 2005.5 | France | CF1930611-05 | AM236938 | GenBank |
| 2005.5 | France | CF1990111-05 | AM236943 | GenBank |
| 2005.5 | France | CF2031011-05 | AM236944 | GenBank |
| 2005 | France | 203029-05 | AM711004 | GenBank |
| 2005 | France | 298098-05 | AM711026 | GenBank |
| 1956 | USA | GA | AY673831 | GenBank |
| 2005.78 | USA | CVB3-MCH | EU144042 | GenBank |
| 2008.4 | China | Fuyang19 | FJ000001 | GenBank |
| 2008.45 | China | GZ803 | FJ357838 | GenBank |
| 2005.52 | Australia | 05.188.2226 | FJ868286 | GenBank |
| 2006.01 | Australia | 05.350.3337 | FJ868287 | GenBank |
| 2006.03 | Australia | 06.006.3430 | FJ868327 | GenBank |
| 2006.04 | Australia | 06.007.1075 | FJ868328 | GenBank |
| 2006.23 | Australia | 06.073.4410 | FJ868329 | GenBank |
| 2008.53 | China | 012/2008TC/SD/CHN | FJ919564 | GenBank |
| 2008.53 | China | 177/2008TC/SD/CHN | FJ919566 | GenBank |
| 2008.86 | China | Beijing0811 | GQ141875 | GenBank |
| 2005 | China | YZ127/SD/CHN/2005/CB3 | GQ246518 | GenBank |
| 2006 | China | AM06HZ/SD/CHN/2006/CB3 | GQ329744 | GenBank |
| 2000 | China | 00190/SD/CHN/2000/CB3 | GQ329745 | GenBank |
| 2000 | China | 00270/SD/CHN/2000/CB3 | GQ329746 | GenBank |
| 2000 | China | 00281/SD/CHN/2000/CB3 | GQ329747 | GenBank |
| 2000 | China | 00284/SD/CHN/2000/CB3 | GQ329748 | GenBank |
| 2000 | China | 00343/SD/CHN/2000/CB3 | GQ329749 | GenBank |
| 2000 | China | 00346/SD/CHN/2000/CB3 | GQ329750 | GenBank |
| 2000 | China | 00359/SD/CHN/2000/CB3 | GQ329751 | GenBank |
| 2000 | China | 00362/SD/CHN/2000/CB3 | GQ329752 | GenBank |
| 2001 | China | 01330/SD/CHN/2001/CB3 | GQ329753 | GenBank |
| 2001 | China | 01338/SD/CHN/2001/CB3 | GQ329754 | GenBank |
| 2008 | China | H156F/SD/CHN/2008/CB3 | GQ329755 | GenBank |
| 2008 | China | H019Y/SD/CHN/2008/CB3 | GQ329756 | GenBank |
| 2002 | China | 02217/SD/CHN/2002/CB3 | GQ329757 | GenBank |
| 2002 | China | 02225/SD/CHN/2002/CB3 | GQ329758 | GenBank |
| 2004 | China | 04327/SD/CHN/2004/CB3 | GQ329759 | GenBank |
| 2004 | China | 04433/SD/CHN/2004/CB3 | GQ329760 | GenBank |
| 2005 | China | 05280/SD/CHN/2005/CB3 | GQ329761 | GenBank |
| 2008 | China | 08132/SD/CHN/2008/CB3 | GQ329762 | GenBank |
| 2008 | China | 08153/SD/CHN/2008/CB3 | GQ329763 | GenBank |
| 2008 | China | 08197/SD/CHN/2008/CB3 | GQ329764 | GenBank |
| 2008 | China | 08201/SD/CHN/2008/CB3 | GQ329765 | GenBank |
| 1994 | China | 94196/SD/CHN/1994/CB3 | GQ329766 | GenBank |
| 2008 | China | 37010408199/SD/CHN/2008/CB3 | GQ329767 | GenBank |
| 2006 | China | SSM-CVB3 | GU109481 | GenBank |
| 2008.56 | China | CB3/SD/sewage/080725 | GU272011 | GenBank |
| 2008.57 | China | CB3/SD/sewage/080729 | GU272012 | GenBank |
| 2009.41 | China | CB3/SD/sewage/090528/1-4H | GU272013 | GenBank |
| 2001 | France | CVB3_CF3109_FRA01 | HF948084 | GenBank |
| 2004 | France | CVB3_CF295067_FRA04 | HF948085 | GenBank |
| 2006 | France | CVB3_CF209062_FRA06 | HF948088 | GenBank |
| 2010.81 | Denmark | Pan_troglodytes | JN979570 | GenBank |
| 2008 | China | 08-2035_verus | JQ042700 | GenBank |
| 1996 | China | 96HN+3/SD/CHN/1996 | JQ364844 | GenBank |
| 2000 | China | 00353/SD/CHN/2000 | JQ364845 | GenBank |
| 2001 | China | 01281/SD/CHN/2001 | JQ364846 | GenBank |
| 2001 | China | 01318/SD/CHN/2001 | JQ364847 | GenBank |
| 2001 | China | 01343/SD/CHN/2001 | JQ364848 | GenBank |
| 2007 | China | 2007AMES.Cao/SD/CHN | JQ364849 | GenBank |
| 2007 | China | 2007AMES.Yang/SD/CHN | JQ364850 | GenBank |
| 2002 | China | 02153/SD/CHN/2002 | JQ364851 | GenBank |
| 2002 | China | 02197/SD/CHN/2002 | JQ364852 | GenBank |
| 2002 | China | 02243/SD/CHN/2002 | JQ364853 | GenBank |
| 2002 | China | 02251/SD/CHN/2002 | JQ364854 | GenBank |
| 2002 | China | 02273/SD/CHN/2002 | JQ364855 | GenBank |
| 2002 | China | 02298/SD/CHN/2002 | JQ364856 | GenBank |
| 2002 | China | 02302/SD/CHN/2002 | JQ364857 | GenBank |
| 2003 | China | 03243/SD/CHN/2003 | JQ364858 | GenBank |
| 2005 | China | 05213/SD/CHN/2005 | JQ364859 | GenBank |
| 2005 | China | 05267/SD/CHN/2005 | JQ364860 | GenBank |
| 2005 | China | 05275/SD/CHN/2005 | JQ364861 | GenBank |
| 2005 | China | 05336/SD/CHN/2005 | JQ364862 | GenBank |
| 2005 | China | 05367/SD/CHN/2005 | JQ364863 | GenBank |
| 2005 | China | 05416/SD/CHN/2005 | JQ364864 | GenBank |
| 2008 | China | 08130/SD/CHN/2008 | JQ364865 | GenBank |
| 2008 | China | 08131/SD/CHN/2008 | JQ364866 | GenBank |
| 2008 | China | 08135C4/SD/CHN/2008 | JQ364867 | GenBank |
| 2008 | China | 08135C5/SD/CHN/2008 | JQ364868 | GenBank |
| 2008 | China | 08180C2/SD/CHN/2008 | JQ364869 | GenBank |
| 2008 | China | 08199/SD/CHN/2008 | JQ364870 | GenBank |
| 2008 | China | 08210/SD/CHN/2008 | JQ364871 | GenBank |
| 2008 | China | 08211/SD/CHN/2008 | JQ364872 | GenBank |
| 2008 | China | 08281/SD/CHN/2008 | JQ364873 | GenBank |
| 2009 | China | 09229/SD/CHN/2009 | JQ364874 | GenBank |
| 1990 | China | 90052/SD/CHN/1990 | JQ364875 | GenBank |
| 1993 | China | 93010/SD/CHN/1993 | JQ364876 | GenBank |
| 1996 | China | 96125/SD/CHN/1996 | JQ364877 | GenBank |
| 1996 | China | 96169/SD/CHN/1996 | JQ364878 | GenBank |
| 1996 | China | 96174/SD/CHN/1996 | JQ364879 | GenBank |
| 1996 | China | 96191/SD/CHN/1996 | JQ364880 | GenBank |
| 1996 | China | 96226/SD/CHN/1996 | JQ364881 | GenBank |
| 1996 | China | 96302/SD/CHN/1996 | JQ364882 | GenBank |
| 2002 | China | 02HZ+4/SD/CHN/2002 | JQ364883 | GenBank |
| 2002 | China | 02HZ+5/SD/CHN/2002 | JQ364884 | GenBank |
| 2010 | China | JNEW100429/SD/CHN/2010 | JQ364885 | GenBank |
| 2000 | Taiwan | CL2000139 | JQ390174 | GenBank |
| 2000 | Taiwan | CL2000-140 | JQ390175 | GenBank |
| 2000 | Taiwan | CL2000141 | JQ390176 | GenBank |
| 2000 | Taiwan | CL2000-142 | JQ390177 | GenBank |
| 2000 | Taiwan | CL2000143 | JQ390178 | GenBank |
| 2005 | Taiwan | 2005-649 | JQ390179 | GenBank |
| 2005 | Taiwan | 2005-812 | JQ390180 | GenBank |
| 2005 | Taiwan | 2005-0927 | JQ390181 | GenBank |
| 2005 | Taiwan | 2005-960 | JQ390182 | GenBank |
| 2005 | Taiwan | 2005-1271 | JQ390183 | GenBank |
| 2005 | Taiwan | 2005-1362 | JQ390184 | GenBank |
| 2005 | Taiwan | 2005-1396 | JQ390185 | GenBank |
| 2005 | Taiwan | 2005-1451 | JQ390186 | GenBank |
| 2000 | Taiwan | 2000758 | JQ390187 | GenBank |
| 2000 | Taiwan | 2000802 | JQ390188 | GenBank |
| 2005 | Taiwan | 2005517 | JQ390189 | GenBank |
| 2001 | Taiwan | 20011693 | JQ390190 | GenBank |
| 2003 | Taiwan | 20031159 | JQ390191 | GenBank |
| 2004 | Taiwan | 20040261 | JQ390192 | GenBank |
| 2004 | Taiwan | 20040263 | JQ390193 | GenBank |
| 2004 | Taiwan | 20040264 | JQ390194 | GenBank |
| 2004 | Taiwan | 20040267 | JQ390195 | GenBank |
| 2004 | Taiwan | 20040269 | JQ390196 | GenBank |
| 2004 | Taiwan | 20040270 | JQ390197 | GenBank |
| 2004 | Taiwan | 20040281 | JQ390198 | GenBank |
| 2005 | Taiwan | 20050957 | JQ390199 | GenBank |
| 2005 | Taiwan | 20051087 | JQ390200 | GenBank |
| 2000 | Taiwan | AFP2000250 | JQ390201 | GenBank |
| 2000 | Taiwan | E2000008 | JQ390202 | GenBank |
| 2005 | Taiwan | E2005351 | JQ390203 | GenBank |
| 2005 | Taiwan | E2005601 | JQ390204 | GenBank |
| 2005 | Taiwan | E2005764 | JQ390205 | GenBank |
| 2005 | Taiwan | E2005852 | JQ390206 | GenBank |
| 2005 | Taiwan | E2005905 | JQ390207 | GenBank |
| 2005 | Taiwan | E20051194 | JQ390208 | GenBank |
| 2005 | Taiwan | E20051274 | JQ390209 | GenBank |
| 1999 | Taiwan | AFP9911003 | JQ390210 | GenBank |
| 1999 | Taiwan | AFP9911004 | JQ390211 | GenBank |
| 2007 | Taiwan | E2007668 | JQ390212 | GenBank |
| 2008 | Taiwan | E20081389 | JQ390213 | GenBank |
| 2008 | Taiwan | E20081390 | JQ390214 | GenBank |
| 2008 | Taiwan | E20081391 | JQ390215 | GenBank |
| 2008 | Taiwan | E20081392 | JQ390216 | GenBank |
| 2008 | Taiwan | E20081393 | JQ390217 | GenBank |
| 2008 | Taiwan | E20081388 | JQ390218 | GenBank |
| 2010 | Taiwan | E2010441 | JQ390219 | GenBank |
| 2009 | India | NIV09001C5LVH7 | JX476161 | GenBank |
| 2009 | India | NIV09001C1LVH1 | JX476162 | GenBank |
| 2009 | India | NIV09001C2LVH2 | JX476163 | GenBank |
| 2009 | India | NIV09001C3LVH4 | JX476164 | GenBank |
| 2009 | India | NIV09001C4LVH5 | JX476165 | GenBank |
| 2010 | India | NIV1021241LV380 | JX476166 | GenBank |
| 2009 | India | NIV0917801LV243 | JX476167 | GenBank |
| 2009 | India | NIV099741LV204 | JX476168 | GenBank |
| 2009 | India | NIV0923491LV157 | JX476169 | GenBank |
| 2009 | India | NIV095181LV33 | JX476170 | GenBank |
| 2009 | India | NIV094331LV26 | JX476171 | GenBank |
| 2008.31 | India | A050D | JX513572 | GenBank |
| 2009.36 | India | A210D | JX513573 | GenBank |
| 2009.41 | India | A219D | JX513574 | GenBank |
| 2009.4 | India | M475 | JX513576 | GenBank |
| 2009.53 | China | A103/KM/09 | JX843810 | GenBank |
| 1997 | Germany | 97-927_1207K | JX946654 | GenBank |
| 1997 | Germany | 97-927_1207R | JX946655 | GenBank |
| 2012.53 | China | CVB3SD2012CHN | JX976770 | GenBank |
| 2011.86 | China | AH30 | KC481610 | GenBank |
| 2008.37 | China | JB14080141 | KC867083 | GenBank |
| 2008.37 | China | JB14080324 | KC867084 | GenBank |
| 2008.37 | China | JB14080351 | KC867085 | GenBank |
| 2008.29 | China | JB14080176 | KC867086 | GenBank |
| 2012.37 | China | JB141230091 | KC867087 | GenBank |
| 2012.45 | China | JB141230178 | KC867088 | GenBank |
| 2012.45 | China | JB141230182 | KC867089 | GenBank |
| 2012.45 | China | JB141230183 | KC867090 | GenBank |
| 2009.45 | India | A79-12a | KF177116 | GenBank |
| 2012 | China | SJZ12-0289F/HeB/CHN/2012 | KF246647 | GenBank |
| 2012 | China | SJZ12-0573T/HeB/CHN/2012 | KF246648 | GenBank |
| 2012 | China | SJZ12-0729F/HeB/CHN/2012 | KF246649 | GenBank |
| 2012 | China | SJZ12-0735F/HeB/CHN/2012 | KF246650 | GenBank |
| 2012 | China | SJZ12-0772T/HeB/CHN/2012 | KF246651 | GenBank |
| 2012 | China | SJZ12-0897F/HeB/CHN/2012 | KF246652 | GenBank |
| 2012 | China | SJZ12-0928F/HeB/CHN/2012 | KF246653 | GenBank |
| 2012 | China | SJZK12-0012F/HeB/CHN/2012 | KF246654 | GenBank |
| 2012 | China | SJZK12-0033F/HeB/CHN/2012 | KF246655 | GenBank |
| 2012 | China | SJZK12-0042F/HeB/CHN/2012 | KF246656 | GenBank |
| 2012 | China | SJZK12-0045F/HeB/CHN/2012 | KF246657 | GenBank |
| 2010 | China | JE002/SD/CHN/12/CB3 | KF246751 | GenBank |
| 2011 | China | JN110617.R2-3 | KF747467 | GenBank |
| 2009 | China | KM06 | KJ020100 | GenBank |
| 1993 | France | 2679 | KJ489414 | GenBank |
| 1949 | Connecticut | Nancy | KJ818302 | GenBank |
| 2012 | China | DH09Y/JS/2012 | KP036479 | GenBank |
| 2012 | China | DH16G/JS/2012 | KP036480 | GenBank |
| 2012 | China | PZ23Y/JS/2012 | KP036481 | GenBank |
| 2008.37 | Indonesia | 2008910445 | KR054737 | GenBank |
| 2009 | India | NIV099351LV53P4 | KR107054 | GenBank |
| 2009 | India | NIV0914321LV141P5 | KR107055 | GenBank |
| 2009 | India | NIV099101LV198P7 | KR107056 | GenBank |
| 2010 | India | NIV1019241LV350P10 | KR107057 | GenBank |
| 2011 | Madagascar | MAD9774-11 | KR232787 | GenBank |
| 2011 | Madagascar | MAD9775-11 | KR232788 | GenBank |
| 2008 | China | 08TC170 | KR362878 | GenBank |
| 2002 | Poland | 1500/PL12/2002 | KU189245 | GenBank |
| 2002 | Poland | 1595/PL20/2002 | KU189246 | GenBank |
| 2004 | Poland | 1792/PL02/2004 | KU189248 | GenBank |
| 2008 | Poland | 2048/PL24/2008 | KU189250 | GenBank |
| 2010.78 | Thailand | EV-B_CV-B3/PMKA0906/THA/2010 | KU574623 | GenBank |
| 2010.12 | Thailand | EV-B_CV-B3/PMKA0219/THA/2010 | KU574624 | GenBank |
| 2012.78 | Russia | SPb_219/12Hel/NAO-17/14/RU | KU841460 | GenBank |
| 2012.78 | Russia | SPb_220/12Hel/NAO-18/14/RU | KU841461 | GenBank |
| 2013.86 | Russia | SPb_5111/13sew/Sar-6/14/RU | KU841462 | GenBank |
| 2008 | China | CB3/2035A | KY286529 | GenBank |
| 2013.62 | Japan | Se4/Fukushima/JPN/2013 | LC012522 | GenBank |
| 2013.7 | Japan | Se5/Fukushima/JPN/2013 | LC012523 | GenBank |
| 2013.78 | Japan | Se6/Fukushima/JPN/2013 | LC012524 | GenBank |
| 2012 | China | S002H-SJZ11-0122T | MH293510 | this study |
| 2012 | China | S143H-SJZKZH12-0032T | MH293511 | this study |
| 2012 | China | S165R-SJZKZH12-0202F | MH293512 | this study |
| 2012 | China | S172R-SJZKZH12-0223F | MH293513 | this study |
| 2012 | China | S176R-SJZKZH12-0232F | MH293514 | this study |
| 2012 | China | S178R-SJZKZH12-0235F | MH293515 | this study |
| 2012 | China | S180R-SJZKZH12-0242F | MH293516 | this study |
| 2016 | China | SD2016-BZ161-CVB3 | MH293517 | this study |
| 2016 | China | SD2016-DZ148-CVB3 | MH293518 | this study |
| 2016 | China | SD2016-DZ155-CVB3 | MH293519 | this study |
| 2016 | China | SD2016-DZ171-CVB3 | MH293520 | this study |
| 2016 | China | SD2016-HZ202H-CVB3 | MH293521 | this study |
| 2016 | China | SD2016-HZ276H-CVB3 | MH293522 | this study |
| 2016 | China | SD2016-JA224-CVB3 | MH293523 | this study |
| 2016 | China | SD2016-JN325H-CVB3 | MH293524 | this study |
| 2016 | China | SD2016-LC100H-CVB3 | MH293525 | this study |
| 2016 | China | SD2016-LC114H-CVB3 | MH293526 | this study |
| 2016 | China | SD2016-LC115H-CVB3 | MH293527 | this study |
| 2016 | China | SD2016-LW041H-CVB3 | MH293528 | this study |
| 2016 | China | SD2016-TA150H-CVB3 | MH293529 | this study |
| 2016 | China | SD2016-TA164H-CVB3 | MH293530 | this study |
| 2016 | China | SD2016-TA178H-CVB3 | MH293531 | this study |
| 2016 | China | SD2016-WF451-CVB3 | MH293532 | this study |
| 2016 | China | SD2016-WF598H-CVB3 | MH293533 | this study |
| 2016 | China | SD2016-WH134-CVB3 | MH293534 | this study |

Table S2. Evolutionary characteristics of coxsackievirus B3 (CV-B3) genotypes based on the entire *VP1* gene.

| Genetype^a^ | Year first isolated(country)^b^ | | Divergence according to the evolutionary model | | | | |
| --- | --- | --- | --- | --- | --- | --- | --- |
|  |  |  | Exponential relaxed clock | |  | Lognormal relaxed clock | |
|  |  |  | Substitution rate(HPD)^c^ | Time of emergence(year [HPD]) |  | Substitution rate(HPD)^c^ | Time of emergence(year [HPD]) |
| A | | 1949(Connecticut) | 6.19(4.77-7.56) | N/A |  | 5.38(4.54-6.27) | N/A |
| B | | 1956(USA) |  | N/A |  |  | N/A |
| C | | 1994(Mainland China) |  | N/A |  |  | N/A |
| D | | 1990(Mainland China) |  | 1984.9(1978.8-1988.6) |  |  | 1984.7(1981.4-1987.4) |
| E | | 1993(France) |  | 1989.8(1984.8-1992.6) |  |  | 1989.4(1986.2-1991.7) |
| F | | 1999(Taiwan of China) |  | 1995.8(1991.2-1998.2) |  |  | 1995.3(1992.7-1997.3) |
| G | | 2000(Taiwan of China) |  | 1996.9(1992.5-1999.1) |  |  | 1995.3(1991.9-1998.8) |
| H | | 2008(India) |  | 2000.7(1993.7-2004.2) |  |  | 1986.4(1974.5-1996.3) |

HPD, 95% highest probability density interval; N/A, no data available due to insufficient sample size (n<2);

^a^ Genotypes were classified based on Fig 1 and distance data between/within groups.

^b^ Year and country based on the GenBank databases for reported strains.

^c^ Substitution rates are expressed as 10^-3^ substitutions per site per year.
